# The Degrees of Freedom Problem in Human Standing Posture: Collective and Component Dynamics

**DOI:** 10.1371/journal.pone.0085414

**Published:** 2014-01-10

**Authors:** Zheng Wang, Ji Hyun Ko, John H. Challis, Karl M. Newell

**Affiliations:** 1 Department of Kinesiology, The Pennsylvania State University, University Park, Pennsylvania, United States of America; 2 Department of Psychiatry and Pediatrics, University of Texas Southwestern Medical Center, Dallas, Texas, United States of America; Faculté de médecine de Nantes, France

## Abstract

The experiment was setup to investigate the coordination and control of the degrees of freedom (DFs) of human standing posture with particular reference to the identification of the collective and component variables. Subjects stood in 3 postural tasks: feet side by side, single left foot quiet stance and single left foot stance with body rocking at the ankle joint in the sagittal plane. All three postural tasks showed very high coherence (∼1) of center of pressure (COP) - center of mass (COM) in the low frequency range. The ankle and hip coherence was mid range (∼.5) with the tasks having different ankle/hip compensatory cophase patterns. The findings support the view that the in-phase relation of the low frequency components of the COP-COM dynamic is the collective variable in the postural tasks investigated. The motions of the individual joints (ankle, knee, hip, neck) and couplings of pair wise joint synergies (e.g., ankle-hip) provide a supporting cooperative role to the preservation of the collective variable in maintaining the COM within the stability region of the base of support (BOS) and minimizing the amount of body motion consistent with the task constraint.

## Introduction

It has long been recognized that human standing posture is not a static action but rather a reflection of a set of continuous small amplitude movements involving instantaneous control of multi-leveled body components (e.g., joints, limb segments, muscles etc.) against gravity in order to sustain the motion of the center of mass (COM) within the stability region of the base of support (BOS) [1]. Clinical studies have shown that postural control can be challenged by neural muscular-articular disorders at the periphery and diseases affecting the central nervous system (CNS) [2]–[7]. However, it is still not established which variable or a set of variables is used to regulate upright stance for healthy individuals. This limitation can be viewed as arising from the challenge of the broader issue introduced by [8], [9]: namely that coordination, control and skill in action is a reflection of a system with many redundant degrees of freedom (DFs).

Research on postural control of upright standing has been mainly driven by two distinct experimental protocols that relate to the DFs question at the *muscular-articular* level posed by [9]. One approach evaluates control mechanisms with respect to the adaptive response of the postural system perturbations. A central finding is that pre-programmed stereotyped ankle and hip strategies are triggered when unexpected external perturbations to the postural system are applied [10]. This approach hypothesizes that postural control requires multiple muscle and joint synergies coordinated in both space and time [11], [12].

The second approach evaluates postural adjustments and fluctuations during quiet stance. The long standing hypothesis assumes that body sway in the sagittal plane is driven by the motion of the system as an inverted pendulum pivoting at the ankle joint whereby the acceleration of COM is proportional to the difference between COM and the net center of pressure (COP_NET_) [1]. Recent studies have reported limitations of the one-segment inverted pendulum model and proposed that postural sway dynamics cannot be fully understood without applying multi-segment models [13]–[18], a position that reopens the centrality of the question as to the DFs problem in postural control. However, a multi-segment model for posture could still incorporate a role for the global variables of COM and COP that is distinct from that of individual joint components and muscular synergies [19]–[21].

According to the hierarchical control theory [8], [9], control structures of the nervous system can be characterized as *action, space, muscular-articular links* (known as *synergies*) and *tone*, in which the level of *space* adapts the output of the *synergy* level to meet the demands of the environmental and task constraints. Bernstein hypothesized that the level to which attention is directed will lead in the control of movement. The dynamic systems approach characterizes the coordination patterns by a *collective variable* [22], [23], that emerges from the interaction between the neural, muscular and segmental *components* involved in perception and action of a self-organized system (e.g., postural control). Given that the goal of postural control is typically not to maintain a certain coordination pattern of multiple joints (e.g., ankle, hip and knee joints) at the *muscular-articular* level, there could be a higher order space variable that is the *collective variable* in the control of upright stance.

The identification of the collective variable [22], [23] is an empirical determination that is within a theoretical context of a complex system of many components (DFs). In a bimanual coordination task, the relative motion (e.g., in-phase or anti-phase depending on the movement frequency) of two index fingers is defined as the collective variable of the system that stabilized at two steady states [24]. In an infant treadmill locomotion study, the collective variable is determined as the infant's alternating step with the assumption that the dynamic condition of one leg provides the information for regulating the initiation and trajectory of the other [25]. In ball juggling, on the other hand, the task constraint is primarily related to the timing with which the hands load and unload the balls [26]. In general, and in spite of relevant theorizing [22], [23], we have limited understanding of the nature of collective variables or order parameters for most movement tasks.

In upright stance, the received interpretation is that COM position is the controlled variable whereas COP is the controlling variable to maintain the COM projection within the BOS. Controversy exists on whether the COP motion is driven by a linear inverted pendulum or a nonlinear multi-linkage system. If body sway is dominated by ankle joint alone, local synergy (i.e., ankle joint motion) should play a significant role in postural control. Otherwise, synergies at the *muscular-articular* level should be mid ranged in activity representing the coordinative and compensatory characteristic DFs of the body [13]–[18]. Our first hypothesis, thereby, is that postural control is realized via a nonlinear system involving multiple component DFs that are modestly coupled and changing their coordination in time.

The COP and COM can be highly coordinated in both postural frameworks of linear and nonlinear systems [1], [13]–[18]. Our position, however, is that the COP-COM coupling can be a candidate for the higher order (i.e., space level) collective variable sustained during quiet stance [22], [23]. Previously, the ankle-hip strategy has been defined as the collective variable of the multi-linkage system [27], [28]. Thus, the second aim of our approach is to identify the macroscopic collective variable of the system with the hypothesis that the central role of the COP-COM dynamic is the variable preserved at the *space* level. Under this assumption, it would be expected that the different upright stances investigated here, side-by-side, single foot and single foot standing with body rocking, would lead to the same COP-COM collective coordination dynamics [22], [23]. This would be reflected in the relative invariance and slow time scale of change of this COP-COM macroscopic relation that contrast to the dynamics of the joint components and muscle synergies over the changing constraints of postural task conditions.

At the *muscular-articular* synergetic level, there is not a one-to-one mapping between the task goal and the joint coordination patterns so that different tasks can induce similar coordination patterns and different coordination dynamics can accommodate the same task demand [22], [23]. This degeneracy in realizing functional equivalence is more likely to be manifest in tasks, such as postural control, where there are many joint space DFs to be regulated [27], [28]. Accordingly, the third hypothesis examined was that joint coupling would change as a function of the standing posture and display more relative variability and a different time scale of change than the COM-COP dynamics. In summary, we investigate the question of the functional roles of the candidate component and collective variables with respect to the relative time scales of their invariance, variance and co-variance under the constraints of different postural tasks.

## Methods

### Subjects and procedures

Ten healthy volunteers (6 male, 4 female; height 169.8±10.69 cm; mass 67.58±14.72 kg) participated in the study. All participants reported no apparent neurological or musculoskeletal dysfunction and performed the postural tasks after giving written informed consent. The experimental protocol was approved by the Institutional Review Board of the Pennsylvania State University.

Prior to the testing, 6 passive markers were attached on the left side of the participant. These anatomical landmarks were: 1) the mandibular condyle, 2) the acromial process, 3) the great trochanter, 4) the lateral femoral condyle, 5) the lateral malleolus, and 6) the head of the fifth metatarsal representing the location of the head, shoulder, hip, knee, ankle, and toe, respectively. A 3-D motion analysis system (Qualysis Track Manager including three ProReflex cameras) was used to capture the kinematic information of all passive markers simultaneously. The kinetic data (force and moment) were collected from a force platform (AMTI, OR6-5-1000). The COP_NET_ time series was derived offline according to [29].

The motion analysis system and the force platform were synchronized for data collection at the sample rate of 100 Hz. Both kinematic and kinetic time series were low-pass filtered by a bi-directional 2^nd^ order Butterworth filter at a 5 Hz cut-off. The respective joint angles were defined according to two adjacent limb segments. For example, the knee joint angle was determined by the angle between the thigh and the shank. The definition of each limb segment (foot, shank, thigh, and trunk) and its mass fraction was based on [29]. The body COM location, in the sagittal plane, was estimated by the weighted average of each individual segment's COM position [30].

Three different postural tasks were tested. They were: 1) feet side-by-side with shoulder width apart quiet standing (SS), 2) left foot only quiet standing (LO), and 3) single left foot standing with body rocking (LR). For the first two stances, the participants were requested to stand as still as possible on the force platform. For the left foot body-rocking task, the participants were instructed to comfortably oscillate their upper-body in the sagittal plane primarily at the ankle joint. Throughout each trial, the participant was instructed to fold his/her arms in front of the chest and focus on a visual target that was 2 m away directly in front of the head. The participant's footprints were traced on the force platform to keep the consistent feet location for each stance. Each participant had two 1 min trials for each stance and 2 min relaxation between trials. Data recording was initiated 5 s after the participant could balance him/herself in the respective posture on the platform. The order of the standing conditions was randomly assigned for each participant.

### Data analysis

To quantify the variation of each joint motion (i.e, neck, hip, knee and ankle), a circular standard deviation (SD) was analyzed as a function of postural task. The coherence and cophase analyses were conducted on both collective (COP-COM) and component variables (ankle-knee, ankle-hip, ankle-neck, knee-hip, knee-neck, and hip-neck coupling) to reveal their coordination dynamic properties. Even though the ankle and hip joints have been shown to be the primary joints for postural control [10], [11], [13], [15], [27], [28], we did not rule out the possible contribution of the other articular synergies.

Coherence is a measure of the variability of the time difference between two signals. The multi-taper spectral analysis was applied for the coherence and cophase calculation. This technique reduces the spectrum estimation bias by obtaining multiple independent estimates from the time series using discrete prolate spheroidal sequences [31]. The choice on the number of leading tapers depends on the sample rate and bandwidth of the time series. On the other hand, introducing more tapers induces poor resolution in the specified frequency bandwidth. Therefore, 5 tapers were applied for all pairs of coherence/cophase analyses, which were computed by:
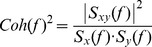
(1)

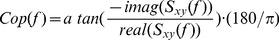
(2)where *S*_x_(*f*) and *S*_y_(*f*) are the power spectral densities of signal *x* and *y* and *S*_xy_(*f*) is the cross power spectral density of the two time series [32].

The coherence provides the extent to which two variables are correlated in the frequency domain and it ranges from 0 (no correlation) to 1 (perfectly correlated). According to the fast fourier transform (FFT), most frequency power of the joint angle, COP and COM time series is less than 0.8016 Hz. Therefore, the results are reported within the range from 0.0167 to 0.8016 Hz with increments of 0.0167 Hz (48 frequency bins). The mean and coefficient of variation (CV) of the coherence were calculated for statistical analyses.

The cophase characterizes the lead-lag relation of two signals as a function of frequency. For example, a 0° cophase indicates an in-phase coupling that two time series simultaneously travel together. On the contrary, an 180° cophase represents an anti-phase coordination that one signal has a half cycle delay to the other (e.g., −180° implies that signal *y* leads *x*). More precisely, if the phase difference is stable and constant over time i.e., phase locked then coherence = 1.0 and if time difference between two signals varies from moment-to-moment then coherence = 0. The descriptive circular statistics (mean and SD) were derived to reveal the cophase patterns for all articular couplings and the COM-COP coordination qualitatively [33].

### Statistical analysis

Due to the circular property of the SD of the joint motion, a Watson-Williams test (analogue of the two sample t-test or the one-way ANOVA for the normally distributed linear data) was conducted for each postural task to reveal the joint main effect. For the coherence, we separated the whole frequency range into 8 equally spaced bins (1. 0.0167–0.1002 Hz, 2. 0.1169–0.2004 Hz, 3. 0.2171–0.3006 Hz, 4. 0.3173–0.4008 Hz, 5. 0.4175–0.5010 Hz, 6. 0.5177–0.6012 Hz, 7. 0.6179–0.7014 Hz, and 8. 0.7181–0.8016 Hz). A one-way ANOVA was conducted on the coherence (mean and CV) of each postural task at each individual frequency bin, respectively. The Tukey post-hoc test was used to determine the differences on all pairs of levels of independent variables. The alpha level was set at 0.05 and effects statistically lower are reported.

## Results

**Figure 1:**
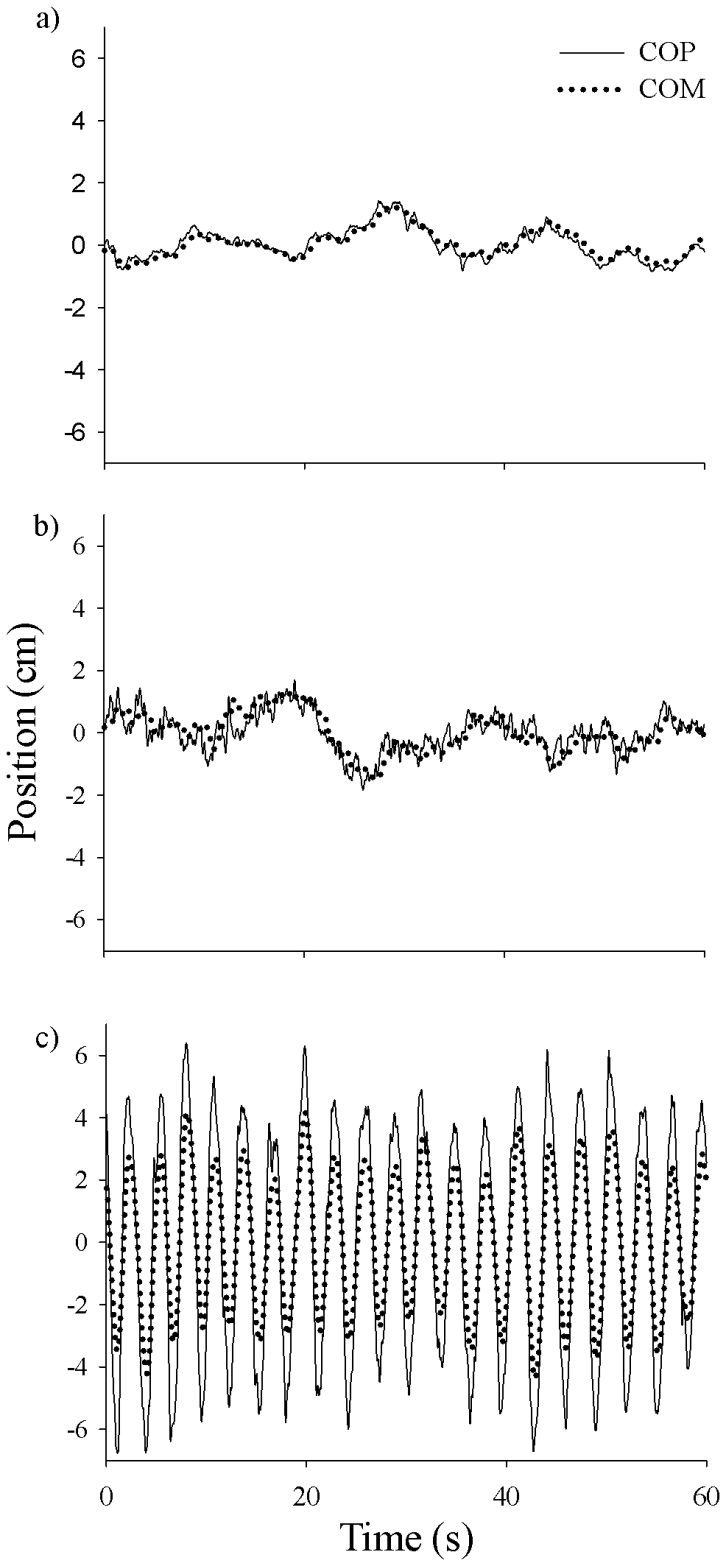
A representative participant's COP and COM motion at different standing conditions: a) Side-by-side; b) single left-foot standing; c) single left-foot standing with body rocking at the ankle joint.

A representative participant's COP and COM motions as a function of stance are presented in Fig. 1. The amount of motion of these two time series gradually increased as a function of postural task. The left foot rocking condition displayed the largest range of motion followed by the single left-foot and the side-by-side quiet stances. Consistent with previous studies [1], the COP motion was larger and more variable than the COM for all standing postures, and the COM and COP tended to move in-phase instantaneously.

Table 1 illustrates the variability of the joint angle as a function of postural task. The Watson-Williams test did not reveal a joint angle main effect for the three tasks. All four joints displayed a small amount of motion that was gradually increased as a function of stance. In fact, the postural stability was sustained through the co-activation of multiple joints rather than through motion at only the ankle joint as the inverted pendulum model implies.

**Table 1:** Mean (M) and standard deviation (SD) of the joint angle (°) as a function of postural task.

	SS		LO		LR	
	M	SD	M	SD	M	SD
Ankle	105.27	0.21	100.00	0.59	99.59	1.18
Hip	151.79	0.30	153.48	0.92	156.10	1.78
Knee	175.30	0.18	172.95	0.57	173.07	0.82
Neck	125.13	0.31	123.08	0.75	123.23	1.26

The joint angle was defined according to its adjacent limb segments.

SS: side-by-side; LO: left foot; LR: left foot body rocking.

**Figure 2:**
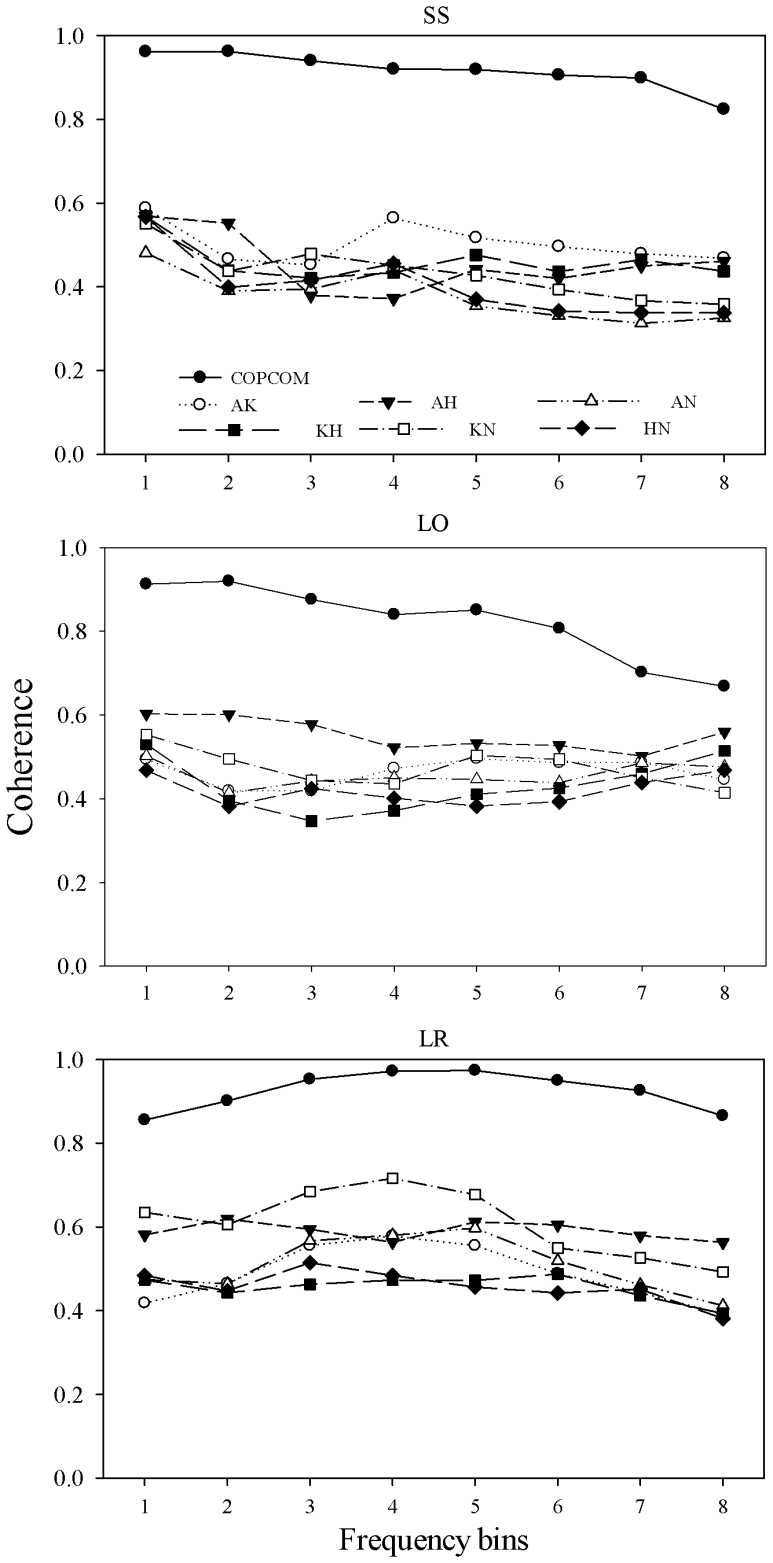
Coherence (group mean) of all pairs of joint angle position (AK: ankle-knee, AH: ankle-hip, AN: ankle-neck, KH: knee-hip, KN: knee-neck, HN: hip-neck) and COP-COM coupling at different standing conditions (SS: side-by-side, LO: single left-foot standing, LR: single-left foot standing with body rocking at the ankle joint). Frequency bins are: 1) 0.0167–0.1002 Hz; 2) 0.1169–0.2004 Hz; 3) 0.2171–0.3006 Hz; 4) 0.3173–0.4008 Hz; 5) 0.4175–0.5010 Hz; 6) 0.5177–0.6012 Hz; 7) 0.6179–0.7014 Hz; and 8) 0.7181–0.8016 Hz.

Fig. 2 shows the coherence of all articular synergies in addition to the COP-COM coordination as a function of frequency. In general, the COP-COM coherence was roughly within the range of 0.8–1, and was larger than that of the joint couplings at each corresponding frequency bin. The patterns of the COP-COM coherence for the side-by-side and single left-foot stances were essentially similar with a steady decrease as a function of frequency. In contrast, the coherence for the left-foot rocking condition gradually increases up to the fifth frequency bin (0.4175–0.5010 Hz) and then gradually decreases.

The one-way ANOVA showed that there was main effect of standing posture across all frequency bins. The post-hoc analysis revealed that the COP-COM coherence of the side-by-side and left-foot rocking conditions is significantly higher than that of all joint couplings for all frequency bins. For the single left-foot stance, the COP-COM coherence was significantly higher than that of the articular synergies from 0.0167 to 0.6012 Hz. There was no significant difference, however, between the COP-COM and the joint couplings at the frequency bin 7 (0.6179–0.7014 Hz). In addition, for the last frequency bin (0.7181–0.8016 Hz), a significant difference was observed only between the COP-COM and the ankle-knee, knee-neck couplings.

**Figure 3:**
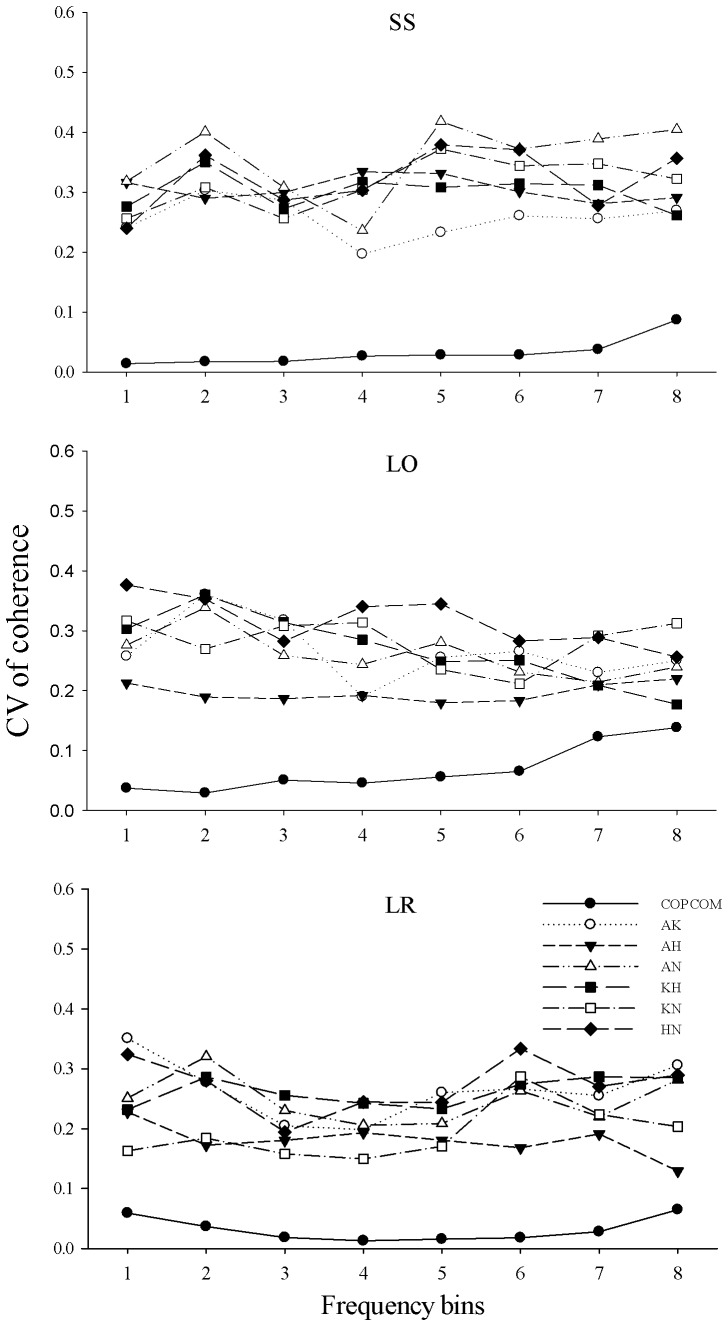
Coefficient of variation of coherence (group mean) for all pairs of joint synergies and COP-COM coupling at different standing conditions. Anatomical abbreviations and frequency bins are as in Fig. 2. Frequency bins are as in Fig. 2.

Fig. 3 shows the CV of the coherence of the COP-COM coupling and articular synergies. In general, the CV of the COP-COM coherence for both side-by-side and single left-foot standings was lower than that of the synergy level couplings and it slightly increased to 0.1 as a function of frequency. For the left-foot rocking task, the CV of the COP-COM coherence was also lower than each joint coupling and it slowly decreased to 0.01 at frequency bin 5 (0.4175–0.5010 Hz) followed by a gradually increase to 0.06. The one-way ANOVA showed a significant main effect of coupling at all frequency bins for all standing conditions. Post-hoc analysis revealed that the CV of the COP-COM coherence was significantly lower than that of all joint couplings with a few exceptions in the single left-foot and left-foot rocking conditions. For example, at frequency bin 1 (0.0167 to 0.1002 Hz), there was no significant difference between the COP-COM and the ankle-hip coupling in the single left foot stance. For the left-foot rocking task, there was no significant difference between the knee-neck coupling and the COP-COM coordination at frequency bin 1 (0.0167 to 0.1002 Hz), 7 (0.6179– 0.7014 Hz) and 8 (0.7181– 0.8016 Hz).

**Figure 4:**
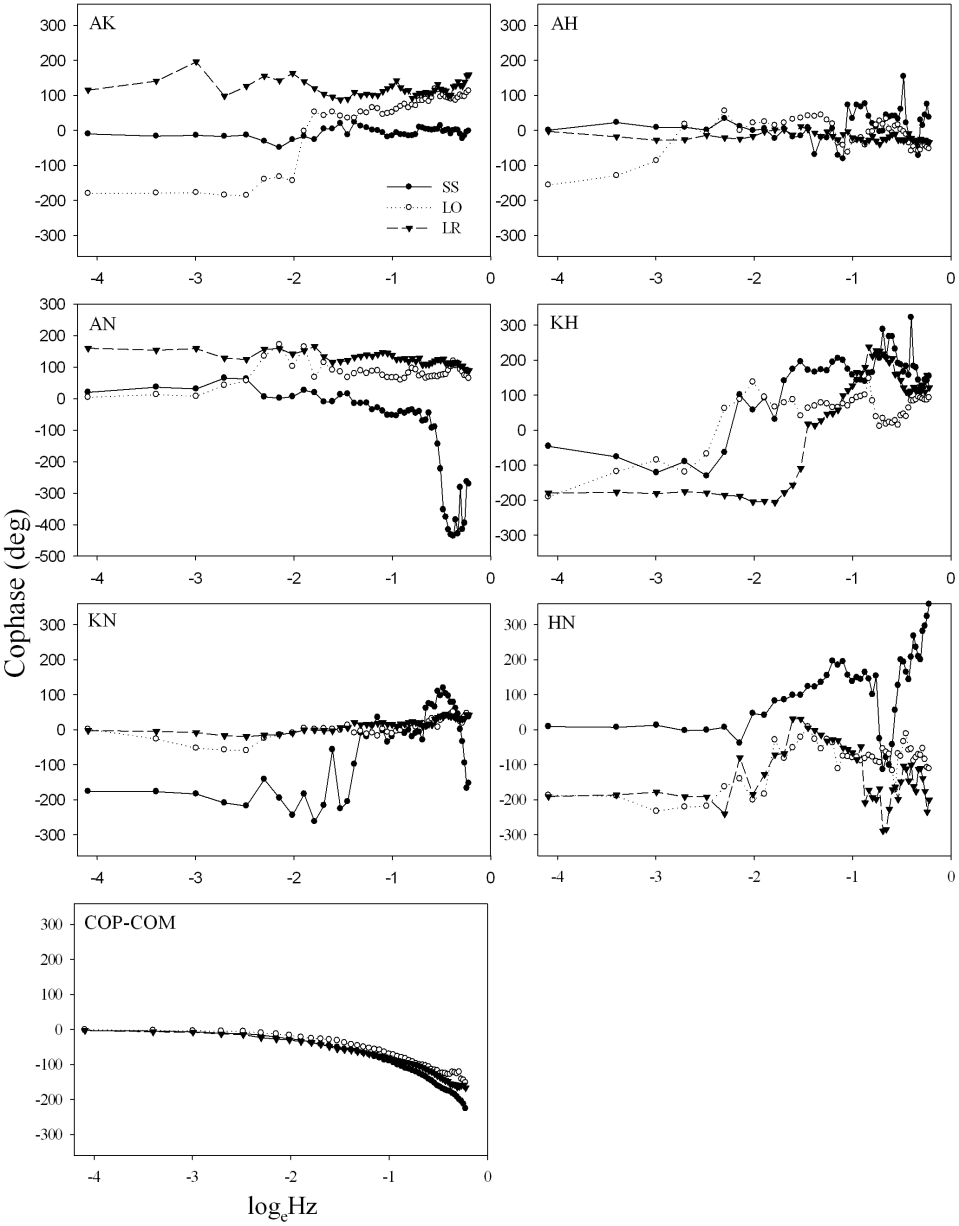
Cophase (group mean) of all pairs of joint synergies and COP-COM coupling at different standing conditions. Anatomical abbreviations are as in Fig. 2.

Fig. 4 illustrates the cophase of all pairs of comparison in both the collective and component levels. The COP-COM cophase patterns were essentially identical for these three postural tasks, in which the COP and COM moved in the same direction instantaneously (cophase ∼0°) from 0.0167 to 0.1169 Hz and gradually decreased at the relatively high frequencies. The negative COP-COM cophase relation indicates that the COM generally leads the COP motion at the high frequencies. On the other hand, there were more cophase variations in the joint couplings as a function of postural stances. For example, the ankle-knee cophase of the left-foot rocking condition was ∼100° as a function of frequency. However, the ankle-neck and knee-neck cophase in the same postural task showed an anti-phase and in-phase coordination, respectively. In addition, certain of the synergy level cophase (e.g., knee-hip, hip-neck couplings) showed larger fluctuations or abrupt changes as a function of frequency.

**Figure 5:**
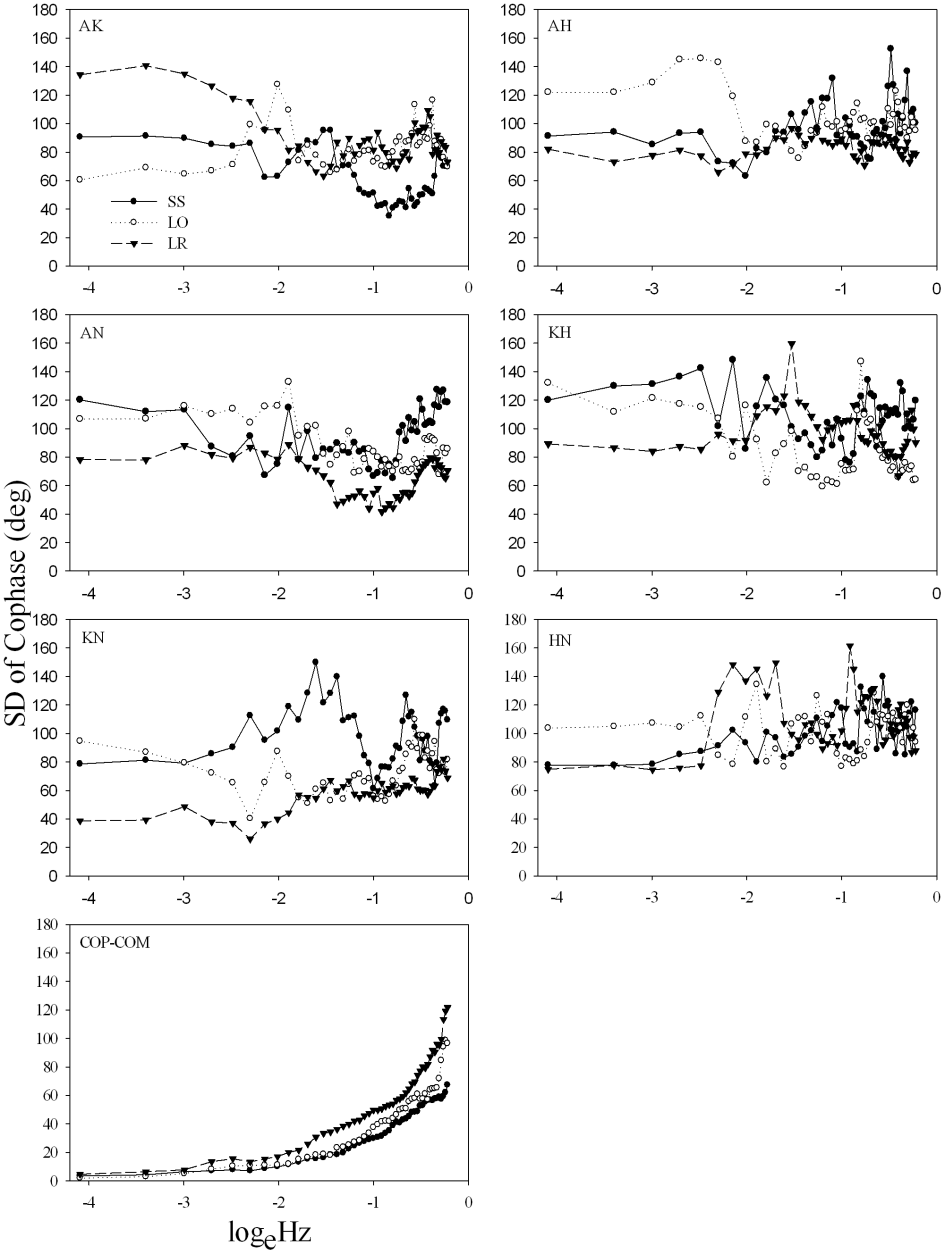
Cophase SD (group mean) of all pairs of joint synergies and COP-COM coupling at different standing conditions. Anatomical abbreviations are as in Fig. 2.

Fig. 5 shows the circular SD of cophase of the collective and component couplings as a function of frequency. The SD of the COP-COM cophase was relatively low (<20°) from 0.0167 to 0.1837 Hz, and then gradually increased at high frequencies. This pattern was consistent for the three standing postures. On the other hand, the cophase SD of the joint couplings was relatively high at the low frequencies (i.e., from 0.0167 to 0.3173 Hz) and the pattern was highly variable as a function of both frequency and standing conditions.

## Discussion

The aim of the present study was to examine the coordination and control of the DFs of human upright standing with particular reference to the identification of the collective and the component variables. Participants were instructed to stand in: 1) feet side-by-side quiet stance, 2) single left foot quiet stance, and 3) single left foot with sagittal plane body rocking. All three postural tasks showed very high coherence (∼1) in the dominant low frequencies of COP-COM dynamics. The coherence of all pairs of joint motions was, however, mid range (∼0.4 – 0.6) with the 3 postural tasks having different compensatory cophase patterns. The findings support the view that the COP-COM in-phase dynamic is the collective variable in the postural tasks studied with the motions of the individual joints (ankle, knee, hip, neck, etc.) and couplings of pairwise joint synergies (e.g., ankle-hip, hip-knee etc.) providing a supporting role to the preservation of the collective variable in maintaining the COM within the BOS.

Postural control is a whole-body movement task that involves multiple joint DFs [13]–[18]. Our results show that all joints (ankle, knee, hip and neck) generated a similar pattern to the relative amount of angular motion across the three postural tasks (Table 1). The hip joint had the largest angular motion across all postural conditions, even in quiet stance. Moreover, the neck also displayed significant angular motion in the three postural tasks probably because of the importance of maintaining head orientation relative to gravity, especially in left-foot rocking condition. These findings on the motions of the individual DFs confirm that postural control involves active control of the multiple individual joint components to maintain body coordination and balance [13]–[18].

Previous studies have proposed that the ankle and hip strategies in quiet stance are two co-existing accessible modes that integrate spatial and temporal movement parameters according to the task and environmental demands [10]–[12]. The dynamic systems approach to posture has proposed that the in/anti-phase coordination between the ankle and hip joints are self-emergent phenomena influenced by the constraints imposed by the informational demands of suprapostural tasks [27]. In addition, it has been shown that the reciprocal relation between the ankle-hip angular acceleration is the primary constraint for quiet stance [15].

However, these studies of postural control are in Bernstein's framework focused at the *muscular-articular* level rather than searching for the potential solution at the collective *space* level. There is evidence that supports the notion of the global control of postural stance implying the existence of the higher order collective variable [19]–[21]. The *space* level adapts the output of the *muscular-articular* level to meet the task and environmental demands. In other words, joint angular motions and their phase relations (including the ankle and hip coordination) are all component and synergetic variables respectively localized at the lower *muscular-articular* level [9].

Given the two main functional goals of postural control are postural orientation and equilibrium the higher-level collective variable candidates could be the head position and COM. Even though it is necessary to maintain an appropriate head orientation while standing, the head predominantly plays a role of the afferent sensor to the CNS because of the localization of both visual and vestibular systems. On the other hand, to sustain the projection of the COM within the BOS has direct linkage to postural stability, even though this task goal, in most of the cases, is realized subconsciously or without the directing of attention to its realization.

In our view, however, motion of the COM by itself does not play a sufficient role to be the collective dynamic variable in postural stance. It has been shown that the human postural control system does not adopt the COM motion as a reference because the COM is non-stationary and tends not to return to its initial position [34], [35]. In addition, COP is a controlling variable driven by the synergies of multiple DFs over the body, generating torques to compensate the deviation of COM [19], [36]. Given this, we interpret that the consistent high coherence of COP-COM in the low frequency ranges to reflect this dynamic as the collective variable for the postural control tasks studied here.

It is significant that for all three tasks the cophase between COP and COM displayed essentially the same pattern whereas joint angle coupling presented different patterns for the three tasks (Fig. 4). This supports the view that as a collective variable, COP-COM coordination is relatively more consistent and on a slower time scale in comparison with the ankle-hip phase relation at the *muscular-articular* level [22], [23]. For example, the ankle-knee and ankle-hip synergies of the left foot stance showed an in-phase to anti-phase transition, even though highly variable (Fig. 5), as a function of frequency. The phase transition at the *muscular-articular* level has been observed and modeled previously [11], [27], [28] and interpreted as the collective variable of postural control. Here, we emphasize that the collective variable is a higher-order parameter that emerges from the interaction of the neural-muscular components. Thus, even though certain muscular-articular synergies showed a phase transition this is not in and of itself sufficient evidence to interpret it as a collective variable for postural control.

The coordination of COP-COM as a collective variable is also evidenced by the highly and systematically correlated patterns illustrated in coherence analysis. Fig. 2 showed that the coherence patterns of side-by-side and single left-foot stances are essentially identical in which COP and COM are highly correlated in lower frequencies (∼0.9) although progressively less coupled at higher frequencies. In the left-foot rocking condition, postural control is a task driven by the natural frequency of the participants' body sway. As a result, COP-COM sustained high coherence from 0.4175 to 0.5010 Hz to accommodate the participants' body rocking frequency (0.400±0.1200 Hz). For comparison, all pairs of joint motion coherence were mid range (∼0.4 – 0.6) with the tasks. The modest level of coupling is probably one of the characteristics of the *muscular-articular* level, in which synergies are adaptively both cooperative and compensatory [9].

**Figure 6:**
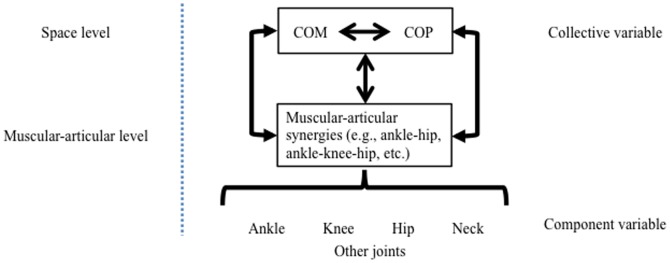
The circular causality for the self-organized postural control system.

Circular causality of the *component* and *collective variables* is the characteristic of a self-organized system with richly coordinated sub-systems at different levels [22], [23]. In the human postural control system, the *collective variable* (i.e., COP-COM) is created by the cooperation of multiple *components* (e.g., ankle, knee, hip and neck, etc.) of the system. Conversely, the preservation of the COP-COM dynamic constrains the behavior of the individual components and lower level synergies as shown in the control schematic of Fig. 6. Indeed, the interconnected synergies at the *muscular-articular* level not only help to maintain the projection of the COM within the BOS but also generate counter-torques at the plantar supporting area against the deviation of the COM [19], [36]. In another words, synergies at the lower level help to preserve the dynamical coordination at the higher level. On the other hand, for maintaining balance, COP-COM needs to be coupled in a certain way to adapt the coordination of the *muscular-articular* level according to the task constraints (e.g., single leg standing, single leg standing with body rocking, etc.).

In summary, we have provided evidence that the COP-COM coordination dynamic could be the collective variable in the set of postural tasks examined here. In this view, the COP-COM dynamic constrains the individual components and local muscular articular synergies of the multiple DFs of the body to satisfy task and environmental constraints. Even though we did not observe a phase transition of COP-COM, a significant feature of fully determining the collective variable of a nonlinear system, it is relevant that studies have reported postural task constraints that induce the anti-phase COP-COM relation [19], [36], [37]. Future studies need to focus on changes of the qualitative COP-COM phase relation as a function of task constraints to provide a fuller examination of the role of collective, synergetic and component variables in postural control.
